# Dietary intake in adults on hemodialysis compared with guideline recommendations

**DOI:** 10.1007/s40620-020-00962-3

**Published:** 2021-02-16

**Authors:** Valeria M. Saglimbene, Guobin Su, Germaine Wong, Patrizia Natale, Marinella Ruospo, Suetonia C. Palmer, Jonathan C. Craig, Juan J. Carrero, Giovanni F. M. Strippoli

**Affiliations:** 1 Faculty of Medicine and Health, Sydney School of Public Health, University of Sydney, Sydney, Australia; 2 Department of Emergency and Organ Transplantation, University of Bari, Piazza Giulio Cesare, 70124 Bari, Italy; 3 National Clinical Research Center for Kidney Disease, State Key Laboratory of Organ Failure Research, Department of Nephrology, Nanfang Hospital, Southern Medical University, Guangzhou City, Guangdong Province China; 4 Department of Nephrology, Guangdong Provincial Hospital of Chinese Medicine, The Second Affiliated Hospital, Guangzhou University of Chinese Medicine, Guangzhou City, Guangdong Province China; 5 Department of Medical Epidemiology and Biostatistics, Karolinska Institutet, Stockholm, Sweden; 6 Department of Renal Medicine, Westmead Hospital, Westmead, Australia; 7 Department of Medicine, University of Otago Christchurch, Christchurch, New Zealand; 8 College of Medicine and Public Health, Flinders University, Adelaide, Australia

**Keywords:** Diet, Nutrition, Haemodialysis, End-stage-kidney-disease

## Abstract

**Background:**

Clinical practice guidelines of dietary management are designed to promote a balanced diet and maintain health in patients undergoing haemodialysis but they may not reflect patients’ preferences. We aimed to investigate the consistency between the dietary intake of patients on maintenance haemodialysis and guideline recommendations.

**Methods:**

Cross-sectional analysis of the DIET-HD study, which included 6,906 adults undergoing haemodialysis in 10 European countries. Dietary intake was determined using the Global Allergy and Asthma European Network (GA^2^LEN) Food Frequency Questionnaire (FFQ), and compared with the European Best Practice Guidelines. Consistency with guidelines was defined as achieving the minimum daily recommended intake for energy (≥ 30 kcal/kg) and protein (≥ 1.1 g/kg), and not exceeding the maximum recommended daily intake for phosphate (≤ 1000 mg), potassium (≤ 2730 mg), sodium (≤ 2300 mg) and calcium (≤ 800 mg).

**Results:**

Overall, patients’ dietary intakes of phosphate and potassium were infrequently consistent with guidelines (consistent in 25% and 25% of patients, respectively). Almost half of the patients reported that energy (45%) and calcium intake (53%) was consistent with the guidelines, while the recommended intake of sodium and protein was consistent in 85% and 67% of patients, respectively. Results were similar across all participating countries. Intake was consistent with all six guideline recommendations in only 1% of patients.

**Conclusion:**

Patients on maintenance haemodialysis usually have a dietary intake which is inconsistent with current recommendations, especially for phosphate and potassium.

**Supplementary Information:**

The online version contains supplementary material available at 10.1007/s40620-020-00962-3.

## Introduction

End-stage kidney disease (ESKD) is a major public health problem affecting more than 2 million people worldwide [1]. The kidney is crucial to nutritional homeostasis. Accordingly, people with ESKD undergoing haemodialysis are advised to modify their dietary intake [2–6]. In particular, clinical practice guidelines recommend restricted phosphate, potassium and sodium intake to avoid elevated serum electrolyte levels and the associated cardiovascular complications [5,^ 7^–13]. Concomitantly, clinical guidelines include recommendations related to sufficient energy and protein intake to avoid malnutrition [2–6]. However, consistency with these stringent recommendations is difficult because of their negative impact on quality of life. Dietary education and support strategies to promote healthy diet and adherence to dietary recommendations are also limited due to resource constraints [14, 15].

Data on dietary intake and consistency with guideline recommendations among patients undergoing haemodialysis are limited to studies with a single-centre design, small sample size and the use of heterogeneous dietary assessment methods. These limitations may preclude generalizability and comparisons across health systems [16–18]. Outside the field of nephrology there is empirical evidence to suggest that consistency with dietary recommendations in the general population or in patients with chronic diseases is associated with better outcomes [19–22].

The purpose of this study was to investigate the consistency between the dietary intake of patients on maintenance haemodialysis and guideline recommendations.

## Materials and methods

### Study design

This is a cross sectional sub-study of the Dietary Intake, Death and Hospitalization in Adults with ESKD Treated with Hemodialysis study (DIET-HD study), a multinational study of dietary intake in adults undergoing haemodialysis [23]. This sub-study is reported according to the Strengthening the Reporting of Observational Studies in Epidemiology (STROBE) guidelines [24].

### Study population

This study includes all DIET-HD patients from the ten European countries (France, Germany, Italy, Hungary, Poland, Portugal, Romania, Spain, Sweden and Turkey) included in the DIET-HD study.

Patients were eligible if they were adults undergoing haemodialysis for at least 90 days. Patients were excluded for﻿ significant neurocognitive disability or medical comorbidity (such as dementia) which would preclude them from understanding the Food Frequency Questionnaire (FFQ) even if assisted, life expectancy less than 6 months or a scheduled kidney transplant within 6 months. Ethics approval was obtained from all relevant institutional ethics committees and the study was conducted in accordance with the Declaration of Helsinki. All patients provided written informed consent.

### Baseline characteristics

Socio-demographic, clinical and dialysis-related data were obtained from a centralized, routinely collected administrative database linked to the participant’s dietary data via their unique provider identification code. All clinical sites used the same standard operating procedures to assess and record the baseline variables of interest.

### Dietary intake assessment

**Fig. 1:**
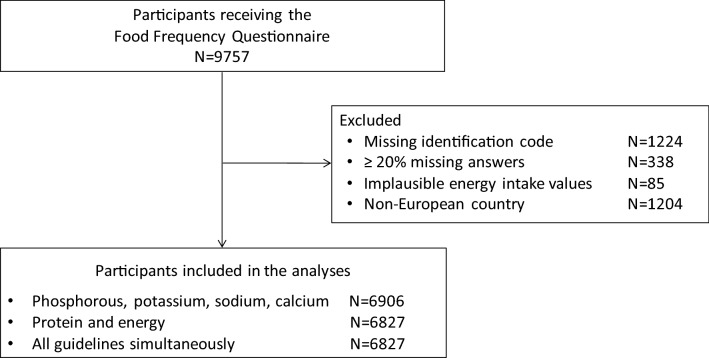
Flow chart of participation

Between January 2014 and January 2015, patients completed, during a dialysis session, the Global Allergy and Asthma European Network (GA^2^LEN) FFQ, which was designed and validated to assess the dietary intake of the past 12 months across countries using a single standardised instrument [25] (Appendix 1). We excluded patients with missing data linkage and those who completed less than 80% of the FFQ or had implausible responses (defined as a log-transformed total energy intake more than three standard deviations from the mean) (Fig. 1). Daily food intake was calculated in grams per day from standard food portion sizes as recommended by the UK’s Food Standards Agency [26]. Macro- and micronutrient intake were derived using the latest available McCance & Widdowson’s Food Composition Tables [27].

## Dietary guidelines

We compared the dietary intake of the study population with the recommendations from the European Best Practice Guidelines (EBPG) [5] (Table S1). These guidelines suggest the following daily target range of intake for nutrients and energy: phosphate 800 to 1000 mg, potassium 1950 to 2730 mg, sodium 2000 to 2300 mg, calcium 500 to 800 mg, protein at least 1.1 g/kg, and energy 30 to 40 kcal/kg. Consistency with each guideline was defined as achieving the minimum daily recommended intake for energy (≥ 30 kcal/kg) and protein (≥ 1.1 g/kg), and not exceeding the maximum recommended daily intake, considered safe to avoid electrolyte imbalances, for phosphate (≤ 1000 mg), potassium (≤ 2730 mg), sodium (≤ 2300 mg) and calcium (≤ 800 mg). The proportion of patients with dietary intakes below, within and above each recommended target was also measured. We also evaluated how many guidelines were met simultaneously.

### Statistical analysis

Baseline demographics were described and dietary intake was estimated overall and by country.

Continuous variables were summarized as mean and standard deviation or median and interquartile range according to their distribution. Categorical variables were summarized as frequencies and proportions. Estimated food (servings per day), energy (kcal) and nutrient (grams per day) intake was analysed as continuous variables and expressed as median and interquartile range. Consistency with each dietary guideline was analysed as a dichotomous variable (achieving or not achieving the minimum daily recommended intake for energy and protein; exceeding or not exceeding the maximum recommended daily intake for phosphate, potassium, sodium and calcium) and a three level variable (below, within and above each target range) and summarized using frequencies and proportions. Analysis of consistency with each dietary guideline included patients with complete data for the guideline of interest. Results were expressed as adjusted odds ratios (OR) and their 95% confidence interval (CI). Analyses were performed in SAS version 9.3 (SAS Institute, Cary, NC).

## Results

Nine thousand seven hundred fifty-seven patients treated with haemodialysis participated in the DIET-HD study. Of these, 6906 (71%) were included in this analysis after exclusion of patients from non-European countries (Argentina) (1204), and those with missing data linkage (1224) or insufficient (338) or implausible (85) FFQ responses (Fig. 1).

The mean age of the study population was 64.4 (standard deviation 14.5) years old, 58% were men, 34% were current or former smokers, 16% engaged in daily physical activity, 86% had reported hypertension, 33% had diabetes, 13% had a prior myocardial infarction, and 9% had a prior history of stroke (Table 1, Table S2).

**Table 1:** Baseline characteristics of patients

Characteristics	N with available data	Mean (SD) or number (%)
Age (years)	6906	
Mean (SD)		64.4 (14.5)
< 65, *n* (%)		3257 (47)
≥ 60, *n* (%)		3649 (53)
Male, *n* (%)	6906	4011 (58)
Country, *n* (%)	6906	
France		221 (3)
Germany		178 (3)
Hungary		554 (8)
Italy		543 (8)
Poland		434 (6)
Portugal		1777 (26)
Romania		1000 (14)
Spain		1041 (15)
Sweden		51 (1)
Turkey		1107 (16)
Current or former smoker, *n* (%)	5688	1918 (34)
Married/Life partner, *n* (%)	5264	3640 (69)
Secondary education or higher, *n* (%)	5506	2524 (46)
Daily physical activity (self-reported), *n* (%)	5613	917 (16)
Body mass index, kg/m^2^ (mean, SD)	6701	
Underweight (< 18.5), *n* (%)		311 (5)
Normal range (18.5–24.9), *n* (%)		2816 (42)
Pre-obese (25.0–29.9), *n* (%)		2286 (34)
Obese (≥ 30.0),* n* (%)		1288 (19)
Hypertension, *n* (%)	6145	5920 (86)
Diabetes Mellitus, *n* (%)	6110	1987 (33)
Congestive heart failure, *n* (%)	6099	1195 (20)
Myocardial infarction, *n* (%)	6065	795 (13)
Stroke, *n* (%)	6058	572 (9)

*SD* standard deviation

### Dietary intake

The median (interquartile range) servings per day of the main food groups were: fruits 2.6 (1.5–4.6), vegetables 3.8 (2.2–6.1), legumes and nuts 0.4 (0.1–0.6), cereals 2.4 (1.3–3.4), dairy 1.4 (0.7–2.4), fish and white meat 0.7 (0.4–1.3), and red meat and meat products 1.1 (0.6 to 1.9). The median daily intake of energy and protein per kg of dry body weight was 28.4 (20.5–38.9) and 1.4 (0.98–1.98), respectively. The median daily intake of minerals was calcium 774 (541–1095) mg, phosphate 1,438 (999–2,047) mg, potassium 3,655 (2,625–5,191) mg and sodium 1337 (929–1907) mg (Table 2). Dietary intake is reported separately by gender in Table S3.

**Table 2:** Daily food, energy, and nutrient intake

Dietary intake	Median (interquartile range)
*Foods (servings/day)*	
Fruit	2.6 (1.5–4.6)
Vegetables	3.8 (2.2–6.1)
Legumes and nuts	0.4 (0.1–0.6)
Cereals	2.4 (1.3–3.4)
Dairy	1.4 (0.7–2.4)
Fish and white meat	0.7 (0.4–1.3)
Red meat and meat products	1.1 (0.6–1.9)
Sweets and sweetened drinks	2.4 (1.1–3.9)
*Energy (kcal/day)*	1937 (14,450–2568)
*Macronutrients (g/day)*	
Carbohydrate	215 (154–295)
Protein	96 (68–131)
Total fat	75 (54–104)
Saturated fat	24 (16–34)
Fibre	12 (8–18)
Total sugar	105 (68–163)
Alcohol	0.7 (0.1–4.7)
*Micronutrients (mg/day)*	
Calcium	774 (541–10,952)
Phosphate	1438 (999–2047)
Potassium	3655 (2625–5191)
Sodium	1337 (929–1907)

*N* = 6903 for fruit, 6905 for vegetables, 6904 for cereals and 6906 for all others

### Consistency with guideline recommendations

**Fig. 2:**
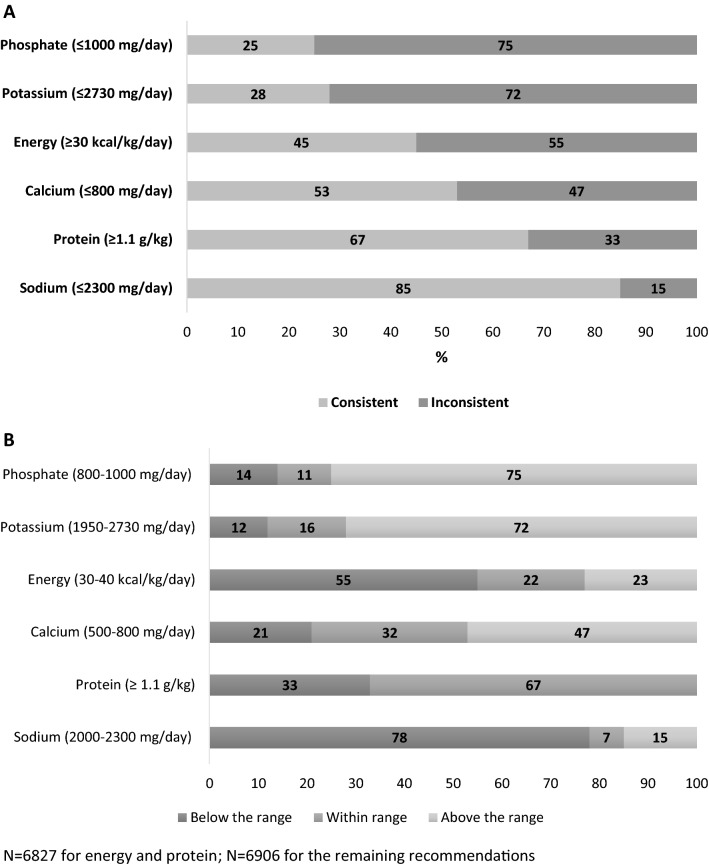
**a** Proportion of patients reporting dietary intake consistent with guidelines (achieving the minimum intake for energy and protein, and not exceeding the maximum intake for phosphate, potassium, sodium and calcium). **b** Proportion of patients reporting dietary intake below, within and above the recommend range of nutrients and energy intake. *N* = 6827 for energy and protein; *N* = 6906 for the remaining recommendations

**Fig. 3:**
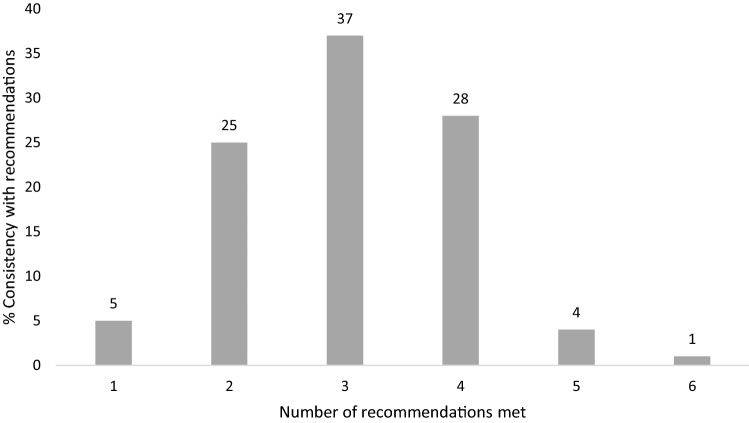
Proportion of patients reporting dietary intake consistent with multiple guideline recommendations (*N* = 6827). The figure shows the percentage of patients that met one or more of the following recommendations: Energy: ≥ 30 kcal/kg/day; Protein: ≥ 1.1 g/kg/day; Phosphate: ≤ 1000 mg/day; Potassium: ≤ 2730 mg/day; Sodium: ≤ 2300 mg/day; Calcium: ≤ 800 mg/day

Respectively, 1731 (25%) and 1914 (28%) patients did not exceed the maximum dietary recommended levels of phosphate (≤ 1000 mg) and potassium (≤ 2730 mg). Dietary intake was consistent with recommended daily energy (≥ 30 kcal/kg, 3084 [45%]) and calcium (≤ 800 mg/day, 3654 [53%]) intake in about half of patients, while the sodium (≤ 2300 mg/day) and protein intake (≥ 1.1 g/kg) was consistent with guidelines in 5848 (85%) and 4598 (67%) of patients, respectively (Fig. 2). The proportion of patients achieving the specific target ranges was very low (Figure S1). Of 6827 patients with available data for the assessment of consistency with all six guideline recommendations simultaneously, 22 (0%) had dietary intakes consistent with zero recommendations, 308 (5%) one, 1731 (25%) two, 2521 (37%) three, 1909 (28%) four, 288 (4%) five, and 48 (1%) had intake consistent with all six recommendations (Fig. 3).

**Fig. 4:**
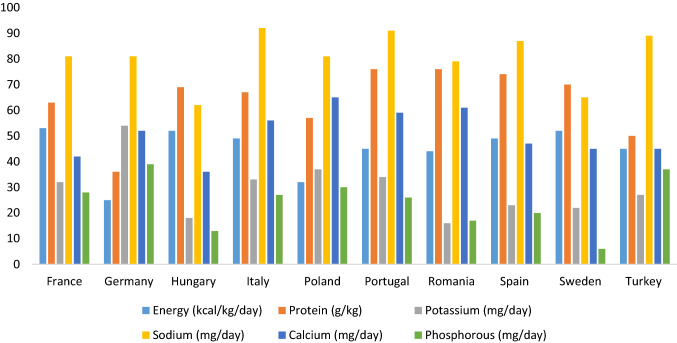
Proportion of patients reporting dietary intake consistent with European Best Practice Guidelines recommendations, by country. The figure shows the percentage of patients that met one or more of the following recommendations: Energy: ≥ 30 kcal/kg/day; Protein: ≥ 1.1 g/kg/day; Phosphate: ≤ 1000 mg/day; Potassium: ≤ 2730 mg/day; Sodium: ≤ 2300 mg/day; Calcium: ≤ 800 mg/day

There was little variation in the consistency with ERPB for sodium (from 62% in Hungary to 92% in Italy) intake among countries, and a larger variation for energy (from 25% in Germany to 53% in France), protein (from 36% in Germany up to 76% in Portugal), potassium (from 16% in Romania up to in 54% Germany), calcium (from 36% in Hungary up to 65% in Poland), and phosphorus (from 6% in Sweden up to 39% in Germany). Overall, the consistency with dietary guidelines varied across countries but followed a similar pattern with lower consistency with phosphate, potassium, energy and calcium and higher consistency with protein and sodium guidelines (Fig. 4, Figure S2, Table S3).

## Discussion

In this study involving 6906 patients undergoing haemodialysis in 10 European countries, the consistency between the reported dietary intake and clinical practice guideline recommendations was generally low. In particular, a high proportion of the study population had dietary intake of phosphate and potassium that exceeded recommendations. In half of the patients, dietary composition exceeded the maximum recommended intake of calcium, while sodium restriction was consistent with guidelines for most patients. Almost 50 and 70% of patients appeared to have dietary total energy and protein content that was consistent with the minimum recommended intake. As expected, the overall consistency was much lower when measured as achieving specific nutrient target ranges (compared with not exceeding the maximum recommended intake or achieving the minimum recommended intake). Consistency with dietary guidelines followed a similar pattern across participating countries. The overall consistency of dietary composition with six renal dietary recommendations occurred in approximately 1% of patients.

Although comparing the dietary intake across different studies is challenging given the variety in study populations and measurement methods used, our finding of generally low consistency of dietary intake with dietary practice guidelines among the haemodialysis population is consistent with previous evidence [16–18, 28, 29] and may be explained by the following. First, the “renal diet” is one of the most difficult-to-attain diets, with a strong focus on nutrients rather than food intake, and strategically difficult to implement in real-life settings [30]. The renal food menus are extremely restrictive, dull, and not enticing. Strict adherence to these diets is known to have a direct negative impact on the overall quality of life of patients on dialysis. Patients report frustration in tracking nutrients, and a perception that ‘there is nothing left to eat’ if they followed the very stringent dietary instructions [15]. Second, the dietary guideline recommendations in haemodialysis are complex, extremely difficult to follow and may require on-going education and guidance from health professionals to achieve consistency of dietary intake. For instance, the most common and simplistic belief for restricting potassium is reducing fruit and vegetable intake. However, potassium is also present in similar amounts in some animal products such as meat and poultry, and in food additives [31]. Cooking methods also play an important role and the combination in which foods are eaten does as well. Boiling reduces the natural potassium content of foods [32], and net potassium absorption in the gut depends on concomitant intake of alkali and fibre [31,^ 33^–35]. In view of the scope and complexity of the renal diet parameters, few nutritional, educational and clinical strategies have shown to be effective in the dialysis and CKD populations [36]. Patients have expressed dissatisfaction with the information they received from health care providers, a great deal of which is unclear or inconsistent [37, 38] and not individualized. Patients are often left unsupported and feel disconnected with their healthcare providers [14,^ 39^, 40]. Third, the evidence that underpins these recommendations is reliant on observational studies and expert opinions without consumer engagement in the guideline development process. Therefore, patients may lack trust in the actual benefits of dietary recommendations and have identified the evaluation of dietary restrictions on health outcomes, including quality of life, as a research priority [41, 42].

Notably, consistency with salt restrictions appeared higher than with recommendations for other nutrients. This could be due to an underestimation of the salt intake as measured by the FFQ or to the large educational campaign in the general population in Europe about the effects of sodium on blood pressure that, after being carried out for several years, may have reached its goal. If this is true, long term educational strategies for other nutrients, such as potassium or phosphorus, could be effective over time in this specific population.

The present study has considerable strengths including the large sample size, the multinational setting and the use of a common dietary assessment method which allowed comparisons of consistency with clinical practice guidelines among European countries. This study also has potential limitations. The dietary intake was self-reported and based on one single measurement. The study relied on a FFQ, which used standard food portion sizes and has been validated against plasma phospholipid fatty acids, but not with other dietary assessment methods, nor in the setting of haemodialysis. Collectively, these limitations may have led to measurement errors and inaccurate absolute dietary intakes. In addition, FFQs may not be sensitive to account for nutrient loss through cooking methods or for the intake of food additives and condiments. This may have resulted in an underestimated intake of nutrients such as phosphate and sodium, and therefore in an increased observed percentage of intake within the safe thresholds. We could only evaluate consistency with dietary guidelines in patients who provided sufficient responses to the FFQ, and extrapolation of results to other dialysis patients should be done with caution. Finally, data on how many centres had a dietary education program or referred patients to dieticians for a structured approach were not available.

These findings of frequently low consistency of dietary content with nutritional recommendations, together with documented lower quality of life with dietary restrictions and the uncertain benefits of single nutrient restrictions [30, 43] suggest that additional evaluation of the evidentiary basis for dietary modification in haemodialysis is warranted. Such an approach is aligned with key research priorities identified by consumers and stakeholders [41, 42]. Our findings of very low consistency with recommendations when evaluating specific nutrient target ranges suggest that dietary guidelines consisting in narrow recommended nutrient levels might not be practical or feasible in real-life settings in which there is no purposive interventional or educational program in the study. Evaluation of whole dietary patterns may be easier due to the lower consistency we see with individual components of dietary content. Besides, the guideline only gives a recommended range of specific values for some nutrients for reference while it should be tailored according to the individual conditions. For instance, in patients with normokalemia and/or good residual renal function, low potassium intake may not be strictly necessary. Of note, the recommended range of specific values in the EBPG is generally consistent with the newly published Kidney Disease Outcome Quality Initiatives (KDOQI) guidelines [44]. It might be interesting to further investigate whether adherence to guidelines is associated with better outcomes in this population.

In conclusion, the dietary intake of patients on haemodialysis is usually inconsistent with guideline recommendations, particularly for recommended levels of phosphate, potassium, calcium and energy intake. Strategies to increase consistency should be evaluated also to support investigations of the impact of dietary recommendations on clinical outcomes and health-related quality of life.

## Supplementary Information

Below is the link to the electronic supplementary material.

Supplementary file1 (PDF 931 KB)
