# Supplementary material for: Dietary intake in adults on hemodialysis compared with guideline recommendations
Source: J Nephrol. 2021 Feb 16;34(6):1999–2007. doi: 10.1007/s40620-020-00962-3 (PMC8610942; doi:10.1007/s40620-020-00962-3)
Supplement: Supplementary file 1 — Supplementary file1 (PDF 931 KB) [file 40620_2020_962_MOESM1_ESM.pdf]

Dietary intake in adults on hemodialysis compared with guideline recommendations

Supplemental material Table of Contents

Table S1. European Best Practice Guidelines for nutrition in hemodialysis

Table S2. Baseline characteristics, by country

Table S3. Daily food, energy, and nutrients intake, by gender

Figure S1. Proportion of patients reporting dietary intake below, within and above the recommend range of nutrients and energy intake

Figure S2. Percentage of patients with nutrients and energy intake below, within and above the recommend range, by country

Appendix 1: The Global Allergy and Asthma European Network (GA2LEN) FFQ questionnaire (Case report form)

Appendix 2: List of clinicians and health care professionals at the participating centres

Table S1. European Best Practice Guidelines for nutrition in hemodialysis

Energy and nutrients	Daily recommended intake
Energy	30-40 kcal/kg
Protein	≥ 1.1 g/kg
Phosphate	800-1000 mg
Potassium	1950-2730 mg
Sodium	2000-2300 mg
Calcium	500-800 mg

Table S2. Baseline characteristics*, by country

Characteristics	France (n=221)	Germany (n=178)	Hungary (n=554)	Italy (n=543)	Poland (n=434)	Portugal (n=1777)	Romania (n=1000)	Spain (n=1041)	Sweden (n=51)	Turkey (n=1107)
Age, years,mean (SD)	70.1 (13.5)	67.1 (13.9)	62.9 (14.4)	67.6 (14.0)	62.3 (13.9)	65.7 (14.6)	58.9 (13.6)	68.2 (14.4)	68.6 (12.9)	61.8 (13.9)
Male,n (%)	131 (59.3)	101 (56.7)	301 (54.3)	343 (63.2)	246 (56.7)	1054 (59.3)	548 (54.8)	673 (64.7)	32 (62.8)	582 (52.6)
Current or former smoke,n (%)r	52 (48.6)	74 (54.8)	158 (28.9)	194 (36.3)	125 (32.0)	431 (29.3)	279 (28.4)	457 (45.3)	22 (45.8)	126 (27.2)
≥Secondary education ,n (%)	61 (59.8)	97 (70.8)	353 (65.2)	303 (57.8)	221 (57.4)	469 (34.4)	541 (54.4)	313 (33.1)	20 (66.7)	146 (30.2)
Daily physical activity ,n (%)	12 (11.8)	17 (12.4)	202 (38.2)	23 (4.3)	43 (10.8)	91 (6.3)	258 (26.4)	169 (16.8)	9 (23.7)	93 (20.4)
Hypertension ,n (%)	99 (89.2)	159 (89.3)	539 (97.3)	421 (77.7)	389 (92.0)	1537 (86.6)	860 (86.0)	945 (92.3)	38 (74.5)	303 (62.2)
Diabetes,n (%)	37 (33.3)	73 (41.0)	201 (36.3)	150 (27.7)	130 (30.7)	574 (32.3)	253 (25.3)	372 (36.3)	22 (43.1)	175 (38.8)
Myocardial infraction,n (%)	21 (30.0)	13 (7.3)	55 (9.9)	73 (13.5)	73 (17.3)	275 (15.5)	67 (6.7)	154 (14.9)	10 (19.6)	54 (12.3)
Stroke,n (%)	5 (7.3)	14 (7.9)	44 (7.9)	19 (3.5)	32 (7.6)	235 (13.2)	93 (9.3)	105 (10.1)	5 (9.8)	20 (4.6)

*Continuous data are expressed as mean (standard deviation) or median (interquartile range). Categorical data are expressed as frequencies (percen

Table S3. Daily food, energy, and nutrients intake, by gender

	Median (interquartile range)	
**Dietary intake**	**Female (N=2895)**	**Male (N=4011)**
**Foods (servings/day)**		
Fruit	2.6 (1.5-4.7)	2.6 (1.5-4.6)
Vegetables	4.0 (2.4-6.2)	3.6 (2.1-5.9)
Legumes and nuts	0.3 (0.1-0.6)	0.4 (0.1-0.7)
Cereals	2.3 (1.3-3.3)	2.4 (1.4-3.6)
Dairy	1.4 (0.6-2.4)	1.4 (0.7-2.4)
Fish and white meat	0.6 (0.3-1.2)	0.7 (0.4-1.3)
Red meat and meat products	0.9 (0.4-1.6)	1.14 (0.6-2.0)
Sweets and sweetened drinks	2.3 (1.1-3.6)	2.4 (1.1-4.0)
**Energy (kcal/day)**	**1864.8 (1395.7-2454.0)**	**1996.1 (1494.4-2634.3)**
**Macronutrients (g/day)**		
Carbohydrate	206.5 (151.0-283.0)	221.8 (156.3-302.4)
Protein	92.0 (64.6-126.8)	99.7 (71.0-134.7)
Total fat	73.2 (52.6-100.7)	76.8 (54.8-106.9)
Saturated fat	23.0 (15.7-32.8)	24.2 (17.1-35.2)
Fibre	12.1 (8.2-17.2)	12.6 (8.6-18.3)
Total sugar	103.2 (67.8-160.1)	106.4 (68.2-164.0)
Alcohol	0.2 (0.0-1.7)	1.6 (0.1-9.5)
**Micronutrients (mg/day)**		
Calcium	753.7 (527.2-1061.7)	785.4 (551.9-1110.6)
Phosphate	1391.3 (961.2-1959.4)	1467.2 (1023.7-2096.0)
Potassium	3568.8 (2552.8-5043.2)	3714.5 (2676.4-5260.1)
Sodium	1292.3 (896.4-1817.0)	1377.6 (949.1-1992.6)

Figure S1. Proportion of patients reporting dietary intake below, within and above the recommend range of nutrients and energy intake

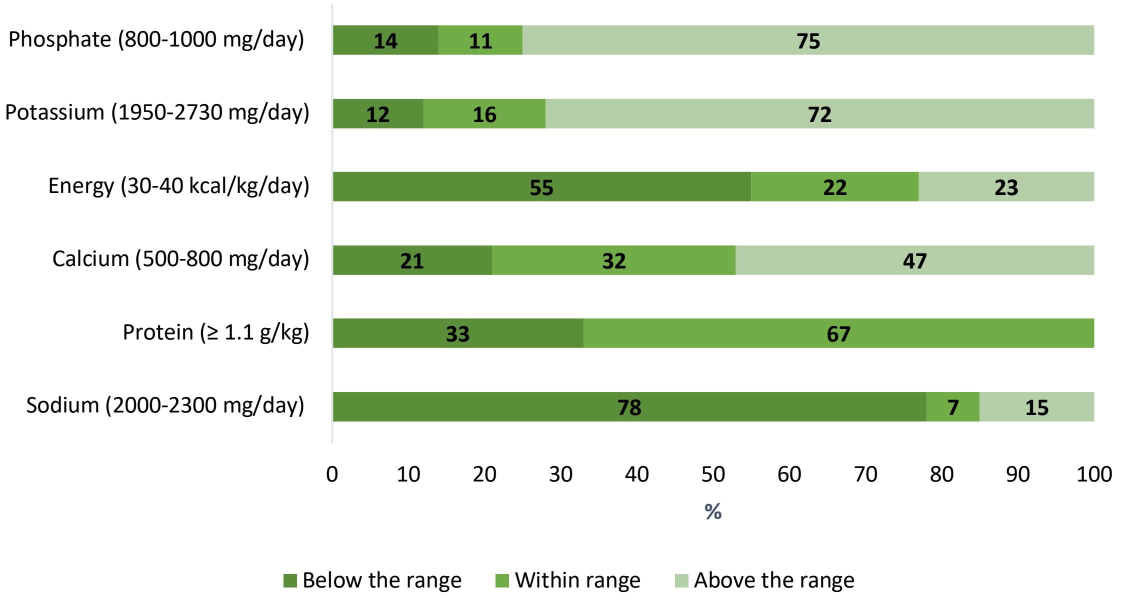

N=6827 for energy and protein; N=6906 for the remaining recommendations

Figure S2. Percentage of patients with nutrients and energy intake below, within and above the recommend range, by country

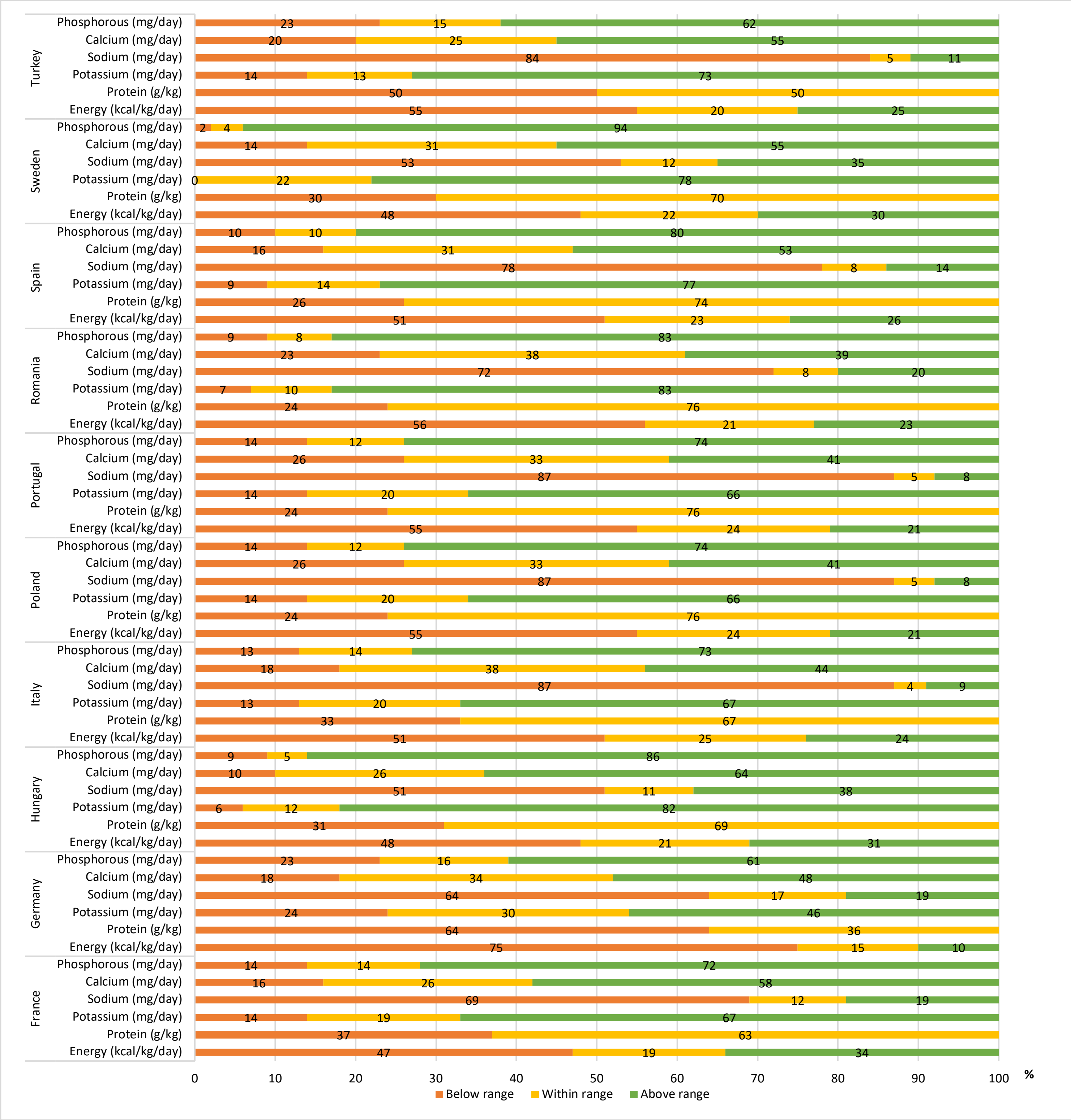

Within the recommended range: phosphate 800 to 1000 mg, potassium 1950 to 2730 mg, sodium 2000 to 2300 mg, calcium 500 to 800 mg, protein at least 1.1 g/kg, and energy 30 to 40 kcal/kg

Appendix 1: The Global Allergy and Asthma European Network (GA2LEN) FFQ questionnaire (Case report form)

DIETARY INTAKE IN HEMODIALYSIS

Promoted by

DIAVERUM

CASE REPORT FORM

IRIMS ID
0 ☐☐☐☐☐☐
1 ☐☐☐☐☐☐
2 ☐☐☐☐☐☐
3 ☐☐☐☐☐☐
4 ☐☐☐☐☐☐
5 ☐☐☐☐☐☐
6 ☐☐☐☐☐☐
7 ☐☐☐☐☐☐
8 ☐☐☐☐☐☐
9 ☐☐☐☐☐☐

VISIT DATE |__|__| |__|__| |__|__|
dd mm yy

DIALYSIS UNIT |__|__|__|__|__|__|__|__|__|__|__|__|__|__|__|__|__|__|__|__|

Dear Participant:

We would like to ask you to complete and return this food frequency questionnaire (FFQ). Please tick (●) in the box to indicate how often, on average, you have eaten the specified amount of each food during the last 12 months. Do not tick more than one box per food.

- Because this FFQ is being used in several countries, YOU WILL BE UNFAMILIAR WITH some of the foods listed in this questionnaire. If you do not eat some of these, please tick the option "Rarely/never".
- If you make a mistake and put a tick in the wrong box just cross through the tick as shown below, and put a tick in the correct box.

EXAMPLE

**Vegetables excluding potatoes (medium serving)**	Rarely/ Never	1-3 times a month	Once a week	2-4 per week	5-6 per week	Once a day	2-3 day	4+ day
Lettuce	○	○	●	● (crossed out)	○	○	○	○

- PLEASE TICK **ONE BOX ONLY** PER LINE AND DO NOT LEAVE FOODS WITHOUT ANSWER.
- For seasonal fruits such as strawberries or grapes, if you eat them about once a week when in season, you should put a tick in the column "once a week".

We thank you very much for your collaboration.

DIET Study Research Team

DIAVERUM

Tick one box for every food to show how often you ate it. Please answer every question, if you are uncertain about how to answer a question then do best you can, but please do not leave a question blank.

1. Bread and rolls

	Rarely/ Never	1-3 times a month	Once a week	2-4 week	5-6 week	Once a day	2-3 day	4+ day
q1p1 Any type of bread	○	○	○	○	○	○	○	○
q1p2 Wholemeal or brown bread (with or without seeds)	○	○	○	○	○	○	○	○
q1p3 White bread (e.g. baguette, rolls, sliced)	○	○	○	○	○	○	○	○
q1p4 Rye bread (any)	○	○	○	○	○	○	○	○
q1p5 Nan bread	○	○	○	○	○	○	○	○
q1p6 Chapatti	○	○	○	○	○	○	○	○
q1p7 Yeast based bread	○	○	○	○	○	○	○	○

2. Breakfast cereals

q2p1 Any breakfast cereals (e.g. oatmeal, wheat germ, cornflakes, Quaker, kasha)	○	○	○	○	○	○	○	○
q2p2 Wheat germ	○	○	○	○	○	○	○	○
q2p3 Quaker (or other oat cereal)	○	○	○	○	○	○	○	○
q2p4 Corn-flakes	○	○	○	○	○	○	○	○
q2p5 All-bran cereals	○	○	○	○	○	○	○	○

3. Semolina

q3p1 Couscous	○	○	○	○	○	○	○	○

4. Pasta (and wheat derived foods)

q4p1 Any pasta (on average)	○	○	○	○	○	○	○	○
q4p2 Plain (refined) pasta (e.g. spaghetti)	○	○	○	○	○	○	○	○
q4p3 Plain wholemeal (unrefined) pasta	○	○	○	○	○	○	○	○

	Rarely/ Never	1-3 times a month	Once a week	2-4 week	5-6 week	Once a day	2-3 day	4+ day
q4p4 Filled pasta (with meat/cheese/vegetables)	○	○	○	○	○	○	○	○
q4p5 Noodles (excluding rice noodles)	○	○	○	○	○	○	○	○

5. Bakery products/desserts

q5p1 Any cakes or pastries (on average)	○	○	○	○	○	○	○	○
q5p2 Cakes (e.g. sponge, chocolate)	○	○	○	○	○	○	○	○
q5p3 Pastries (e.g. croissants)	○	○	○	○	○	○	○	○
q5p4 Rolls (with/without stuffing)	○	○	○	○	○	○	○	○
q5p5 Muffins	○	○	○	○	○	○	○	○
q5p6 Doughnuts, buns (plain or filled)	○	○	○	○	○	○	○	○
q5p7 Rice pudding	○	○	○	○	○	○	○	○
q5p8 Cheese cake	○	○	○	○	○	○	○	○
q5p9 Pancakes	○	○	○	○	○	○	○	○
q5p10 Plain biscuits (with no fillings or cream)	○	○	○	○	○	○	○	○

6. Rice

q6p1 Rice (any)	○	○	○	○	○	○	○	○
q6p2 White rice	○	○	○	○	○	○	○	○
q6p3 Brown/wholemeal (unrefined) rice	○	○	○	○	○	○	○	○
q6p4 Rice noodles	○	○	○	○	○	○	○	○

7. Sugar & jam

q7p1 Table sugar (white)	○	○	○	○	○	○	○	○
q7p2 Jam	○	○	○	○	○	○	○	○

	Rarely/ Never	1-3 times a month	Once a week	2-4 week	5-6 week	Once a day	2-3 day	4+ day
q7p3 Marmalade	○	○	○	○	○	○	○	○
q7p4 Honey	○	○	○	○	○	○	○	○

8. Sugar products excluding chocolate

q8p1 Any sweets or bonbons	○	○	○	○	○	○	○	○
q8p2 Boiled sweets, toffees, caramels	○	○	○	○	○	○	○	○
q8p3 Mixed candies	○	○	○	○	○	○	○	○
q8p4 Cereal bars, flapjacks/fruit bar	○	○	○	○	○	○	○	○
q8p5 Water ice (lolly ice)	○	○	○	○	○	○	○	○

9. Chocolate

q9p1 Chocolates (any)	○	○	○	○	○	○	○	○
q9p2 Chocolate snack bars (e.g. Mars bar)	○	○	○	○	○	○	○	○
q9p3 Dark chocolate	○	○	○	○	○	○	○	○
q9p4 Milk chocolate	○	○	○	○	○	○	○	○

10. Vegetable oils

q10p1 Vegetable oil (blended, any)	○	○	○	○	○	○	○	○
q10p2 Sunflower oil	○	○	○	○	○	○	○	○
q10p3 Olive oil	○	○	○	○	○	○	○	○
q10p4 Extra virgin olive oil	○	○	○	○	○	○	○	○
q10p5 Palm oil	○	○	○	○	○	○	○	○

11. Margarine and lipids of mixed origin

q11p1Any margarine or spread (excluding soya spread)	○	○	○	○	○	○	○	○
q11p2 Low-fat margarine	○	○	○	○	○	○	○	○

DIAVERUM

	Rarely/ Never	1-3 times a month	Once a week	2-4 week	5-6 week	Once a day	2-3 day	4+ day
q11p3 Normal margarine	○	○	○	○	○	○	○	○
q11p4 Blended spreads	○	○	○	○	○	○	○	○
q11p5 Soya-based margarine or spreads	○	○	○	○	○	○	○	○
q11p6 Any margarines or vegetable spreads fortified with omega-3	○	○	○	○	○	○	○	○

12. Butter and animal fats

q12p1 Any butter	○	○	○	○	○	○	○	○
q12p2 Low/reduced fat butter	○	○	○	○	○	○	○	○
q12p3 Normal butter	○	○	○	○	○	○	○	○
q12p4 Lard	○	○	○	○	○	○	○	○

13. Nuts

q13p1 Any nuts	○	○	○	○	○	○	○	○
q13p2 Peanuts	○	○	○	○	○	○	○	○
q13p3 Cashew nuts	○	○	○	○	○	○	○	○
q13p4 Almonds	○	○	○	○	○	○	○	○
q13p5 Walnuts	○	○	○	○	○	○	○	○

14. Legumes

q14p1Any legumes	○	○	○	○	○	○	○	○
q14p2 Kidney (red), black beans	○	○	○	○	○	○	○	○
q14p3 Lentils	○	○	○	○	○	○	○	○
q14p4 Chickpeas (also hummus)	○	○	○	○	○	○	○	○
q14p5 Cluster beans (guar)	○	○	○	○	○	○	○	○

DIAVERUM

	Rarely/ Never	1-3 times a month	Once a week	2-4 week	5-6 week	Once a day	2-3 day	4+ day
q14p6 French beans (string beans)	◯	◯	◯	◯	◯	◯	◯	◯
q14p7 Fava beans	◯	◯	◯	◯	◯	◯	◯	◯
q14p8 Soya beans	◯	◯	◯	◯	◯	◯	◯	◯

15. Vegetables excluding potatoes

q15p1 Any vegetables (excluding potatoes)	◯	◯	◯	◯	◯	◯	◯	◯
q15p2 Lettuce	◯	◯	◯	◯	◯	◯	◯	◯
q15p3 Spinach (including lamb's quarters)	◯	◯	◯	◯	◯	◯	◯	◯
q15p4 Chard	◯	◯	◯	◯	◯	◯	◯	◯
q15p5 Fenugreek	◯	◯	◯	◯	◯	◯	◯	◯
q15p6 Wild greens (e.g. purslane, watercress)	◯	◯	◯	◯	◯	◯	◯	◯
q15p7 Okra	◯	◯	◯	◯	◯	◯	◯	◯
q15p8 Tomato	◯	◯	◯	◯	◯	◯	◯	◯
q15p9 Aubergine	◯	◯	◯	◯	◯	◯	◯	◯
q15p10 Courgette	◯	◯	◯	◯	◯	◯	◯	◯
q15p11 Sweet peppers (e.g. red, green, yellow)	◯	◯	◯	◯	◯	◯	◯	◯
q15p12 Cucumber	◯	◯	◯	◯	◯	◯	◯	◯
q15p13 Bitter melon (Karela)	◯	◯	◯	◯	◯	◯	◯	◯
q15p14 Carrots	◯	◯	◯	◯	◯	◯	◯	◯
q15p15 Parsnip	◯	◯	◯	◯	◯	◯	◯	◯
q15p16 Turnip or Swede	◯	◯	◯	◯	◯	◯	◯	◯
q15p17 Artichokes	◯	◯	◯	◯	◯	◯	◯	◯
q15p18 Radish	◯	◯	◯	◯	◯	◯	◯	◯
q15p19 Beetroot	◯	◯	◯	◯	◯	◯	◯	◯
q15p20 Celery	◯	◯	◯	◯	◯	◯	◯	◯
q15p21 Coleslaw	◯	◯	◯	◯	◯	◯	◯	◯

	Rarely/ Never	1-3 times a month	Once a week	2-4 week	5-6 week	Once a day	2-3 day	4+ day
q15p22 Sweet Corn	◯	◯	◯	◯	◯	◯	◯	◯
q15p23 Asparagus	◯	◯	◯	◯	◯	◯	◯	◯
q15p24 Herbs (e.g. mint, fennel, chive, basil, dill, coriander, parsley)	◯	◯	◯	◯	◯	◯	◯	◯
q15p25 Leek	◯	◯	◯	◯	◯	◯	◯	◯
q15p26 White/other mushrooms	◯	◯	◯	◯	◯	◯	◯	◯
q15p27 Onion	◯	◯	◯	◯	◯	◯	◯	◯
q15p28 Garlic	◯	◯	◯	◯	◯	◯	◯	◯
q15p29 Cauliflower	◯	◯	◯	◯	◯	◯	◯	◯
q15p30 Pumpkin	◯	◯	◯	◯	◯	◯	◯	◯
q15p31 Brussels sprouts	◯	◯	◯	◯	◯	◯	◯	◯
q15p32 Peas (green)	◯	◯	◯	◯	◯	◯	◯	◯
q15p33 Broccoli	◯	◯	◯	◯	◯	◯	◯	◯
q15p34 Cabbage (e.g. white, green red, Savoy)	◯	◯	◯	◯	◯	◯	◯	◯
q15p35 Stuffed vegetables (e.g. vine/green leaves with rice or meat)	◯	◯	◯	◯	◯	◯	◯	◯
q15p36 Pickled vegetables (e.g. cucumber, radish, cabbage)	◯	◯	◯	◯	◯	◯	◯	◯
q15p37 Ginger (e.g. in savoury and sweet dishes, in infusion)	◯	◯	◯	◯	◯	◯	◯	◯

16. Starchy roots or potatoes

q16p1 Potatoes (on average, in all forms)	◯	◯	◯	◯	◯	◯	◯	◯
q16p2 Mashed potatoes	◯	◯	◯	◯	◯	◯	◯	◯
q16p3 Baked/roasted/casserole	◯	◯	◯	◯	◯	◯	◯	◯
q16p4 Chips/French fries	◯	◯	◯	◯	◯	◯	◯	◯
q16p5 In salads	◯	◯	◯	◯	◯	◯	◯	◯
q16p6 Potato dumpling, bread dumpling, gnocchi	◯	◯	◯	◯	◯	◯	◯	◯
q16p7 Potato tortilla (omelette)	◯	◯	◯	◯	◯	◯	◯	◯
q16p8 Sweet potato	◯	◯	◯	◯	◯	◯	◯	◯

17. Fruits

	Rarely/ Never	1-3 times a month	Once a week	2-4 week	5-6 week	Once a day	2-3 day	4+ day
q17p1 Fresh fruits (any)	◯	◯	◯	◯	◯	◯	◯	◯
q17p2 Apple	◯	◯	◯	◯	◯	◯	◯	◯
q17p3 Pear	◯	◯	◯	◯	◯	◯	◯	◯
q17p4 Avocado	◯	◯	◯	◯	◯	◯	◯	◯
q17p5 Mango	◯	◯	◯	◯	◯	◯	◯	◯
q17p6 Apricot	◯	◯	◯	◯	◯	◯	◯	◯
q17p7 Nectarine	◯	◯	◯	◯	◯	◯	◯	◯
q17p8 Peach	◯	◯	◯	◯	◯	◯	◯	◯
q17p9 Plum	◯	◯	◯	◯	◯	◯	◯	◯
q17p10 Cherries	◯	◯	◯	◯	◯	◯	◯	◯
q17p11 Rhubarb	◯	◯	◯	◯	◯	◯	◯	◯
q17p12 Berries (e.g. blueberry, strawberry, blackcurrants, blackberry raspberry)	◯	◯	◯	◯	◯	◯	◯	◯
q17p13Banana	◯	◯	◯	◯	◯	◯	◯	◯
q17p14 Melon/ Watermelon	◯	◯	◯	◯	◯	◯	◯	◯
q17p15 Grape	◯	◯	◯	◯	◯	◯	◯	◯
q17p16 Squeezed fresh fruit	◯	◯	◯	◯	◯	◯	◯	◯
q17p17 Pineapple	◯	◯	◯	◯	◯	◯	◯	◯
q17p18 Kiwi	◯	◯	◯	◯	◯	◯	◯	◯
q17p19 Lemon	◯	◯	◯	◯	◯	◯	◯	◯
q17p20 Orange	◯	◯	◯	◯	◯	◯	◯	◯
q17p21 Mandarin/Tangerine	◯	◯	◯	◯	◯	◯	◯	◯
q17p22 Grapefruit	◯	◯	◯	◯	◯	◯	◯	◯
q17p23 Tinned fruits	◯	◯	◯	◯	◯	◯	◯	◯
q17p24 Raisin, sultana	◯	◯	◯	◯	◯	◯	◯	◯
q17p25 Fig	◯	◯	◯	◯	◯	◯	◯	◯
q17p26 Prune	◯	◯	◯	◯	◯	◯	◯	◯
q17p27 Olives (e.g. black, green)	◯	◯	◯	◯	◯	◯	◯	◯
q17p28 Dates	◯	◯	◯	◯	◯	◯	◯	◯

18. Fruit juices (1 glass 200 ml)

	Rarely/ Never	1-3 times a month	Once a week	2-4 week	5-6 week	Once a day	2-3 day	4+ day
q18p1 Concentrated juice, with sugar	○	○	○	○	○	○	○	○
q18p2 Concentrated juice, without sugar (with sweetener)	○	○	○	○	○	○	○	○

19. Non-alcoholic beverages (1 glass 200ml)

q19p1 Carbonated/soft drinks with sugar	○	○	○	○	○	○	○	○
q19p2 Carbonated/soft drinks with artificial sweetener	○	○	○	○	○	○	○	○
q19p3 Tap water	○	○	○	○	○	○	○	○
q19p4 Mineral water (e.g. still or sparkling)	○	○	○	○	○	○	○	○

20. Tea/coffee

q20p1 Black tea (any)	○	○	○	○	○	○	○	○
q20p2 Coffee (instant or ground)	○	○	○	○	○	○	○	○
q20p3 Greek (Turkish) Coffee	○	○	○	○	○	○	○	○
q20p4 Green tea	○	○	○	○	○	○	○	○
q20p5 Peppermint tea	○	○	○	○	○	○	○	○
q20p6 Other herbal infusions	○	○	○	○	○	○	○	○

21. Beer (1/2 pint or 1 glass 200 ml)

q21p1 Beer (any)	○	○	○	○	○	○	○	○

22. Wine (1 glass125 ml)

q22p1 Any wine	○	○	○	○	○	○	○	○
q22p2 Red wine	○	○	○	○	○	○	○	○
q22p3 White wine	○	○	○	○	○	○	○	○
q22p4 Rose wine	○	○	○	○	○	○	○	○

DIAVERUM

23. Other alcoholic beverages (1 glass 50 ml)

	Rarely/ Never	1-3 times a month	Once a week	2-4 week	5-6 week	Once a day	2-3 day	4+ day
q23p1 Fortified wines (Liqueurs) (e.g. Sherry, port, Madeira)	○	○	○	○	○	○	○	○
q23p2 Spirits (e.g. whisky, vodka, rum, gin)	○	○	○	○	○	○	○	○

24. Red meat and meat products

q24p1 Any red meat (e.g. beef, veal, lamb, pork, game)	○	○	○	○	○	○	○	○
q24p2 Hot/cold roast beef, boiled beef, beef steak, fillet, loin	○	○	○	○	○	○	○	○
q24p3 Beef burger (hamburger)	○	○	○	○	○	○	○	○
q24p4 Minced beef meat (e.g chilli con carne, Bolognese sauce, meatballs)	○	○	○	○	○	○	○	○
q24p5 Beef meat in stew, casserole, in curry	○	○	○	○	○	○	○	○
q24p6 Pork cutlet, chop, steak, fillet, loin, pork ribs, minced	○	○	○	○	○	○	○	○
q24p7 Meat pies	○	○	○	○	○	○	○	○
q24p8 Sausages	○	○	○	○	○	○	○	○
q24p9 Veal	○	○	○	○	○	○	○	○
q24p10 Small game (e.g. rabbit, goat, pheasant, duck)	○	○	○	○	○	○	○	○
q24p11 Other game (e.g. deer, moose)	○	○	○	○	○	○	○	○
q24p12 Lamb (e.g. in stews, kebabs)	○	○	○	○	○	○	○	○
Smoked/cured meat (3 slices)								
q24p13 Cured pork (cold or hot-cooked)	○	○	○	○	○	○	○	○
q24p14 Gammon, ham (e.g. Serrano, prosciutto)	○	○	○	○	○	○	○	○
q24p15 Dried cured sausages (chorizo, salchichon, salami)	○	○	○	○	○	○	○	○

	Rarely/ Never	1-3 times a month	Once a week	2-4 week	5-6 week	Once a day	2-3 day	4+ day
q24p16 Frankfurter	◯	◯	◯	◯	◯	◯	◯	◯
q24p17 Bacon, bacon cubes	◯	◯	◯	◯	◯	◯	◯	◯
q24p18 Smoked lamb	◯	◯	◯	◯	◯	◯	◯	◯
q24p19Smoked game (any)	◯	◯	◯	◯	◯	◯	◯	◯

25. Poultry

q25p1 Any poultry with skin	◯	◯	◯	◯	◯	◯	◯	◯
q25p2 Any poultry without skin	◯	◯	◯	◯	◯	◯	◯	◯
Fresh (un-smoked)								
q25p3 Chicken (e.g. boiled, roasted, chicken burgers)	◯	◯	◯	◯	◯	◯	◯	◯
q25p4 Chicken (e.g. stews or casserole)	◯	◯	◯	◯	◯	◯	◯	◯
q25p5 Turkey (e.g. roasted, boiled, strips)	◯	◯	◯	◯	◯	◯	◯	◯
Smoked or cured poultry								
q25p6 Any smoked/cured poultry	◯	◯	◯	◯	◯	◯	◯	◯

26. Offal

q26p1 Liver (eg panita), pates, potted meat	◯	◯	◯	◯	◯	◯	◯	◯
q26p2 Other offal (e.g. tongue, brain, heart, kidney, tripe)	◯	◯	◯	◯	◯	◯	◯	◯

27. Fish and seafood

q27p1 Any fish or seafood (fresh, tinned, smoked, etc)	◯	◯	◯	◯	◯	◯	◯	◯
q27p2 Fresh fatty fish (e.g. salmon, tuna, trout, anchovy, herring, mackerel, sardine, gravalex, eel)	◯	◯	◯	◯	◯	◯	◯	◯

	Rarely/ Never	1-3 times a month	Once a week	2-4 week	5-6 week	Once a day	2-3 day	4+ day
q27p3 Fresh white fish (e.g. hake/burbot, cod, haddock, plaice, whiting)	○	○	○	○	○	○	○	○
q27p4 Other fresh fish/seafood products (e.g. taramasalata)	○	○	○	○	○	○	○	○
q27p5 Fresh Crustaceans and molluscs (e.g. mussel, crab, calamari, octopus, cuttlefish, [illegible]	○	○	○	○	○	○	○	○
q27p6 Cured or smoked fatty fish (sardines, tuna, salmon, kipper)	○	○	○	○	○	○	○	○
q27p7 Cured or smoked white fish (e.g. cod, bacalhau)	○	○	○	○	○	○	○	○
q27p8 Tinned fish (sardine, tuna or salmon)	○	○	○	○	○	○	○	○
q27p9 Tinned crustaceans and molluscs (e.g. mussel, crab, calamari, octopus, cuttlefish, shrimp, clam)	○	○	○	○	○	○	○	○

28. Eggs (from hen)

q28p1 Eggs (any, on average)	○	○	○	○	○	○	○	○
q28p2 Eggs (fried/poached/boiled/hard boiled/in sandwiches)	○	○	○	○	○	○	○	○
q28p3 Egg-based savoury dishes	○	○	○	○	○	○	○	○
q28p4 Egg-based desserts (e.g. Egg cakes, tarts, egg and nuts sweets)	○	○	○	○	○	○	○	○

29. Milk, dairy and soya

q29p1 Milk (any, excluding soya)	○	○	○	○	○	○	○	○
**Cow milk**								
q29p2 Full-fat milk	○	○	○	○	○	○	○	○
q29p3 Semi-skimmed milk	○	○	○	○	○	○	○	○
q29p4 Skimmed milk	○	○	○	○	○	○	○	○
q29p5 Milk fortified with omega 3 fatty acids	○	○	○	○	○	○	○	○
q29p6 Yogurt (any type including fromage)	○	○	○	○	○	○	○	○
	○	○	○	○	○	○	○	○

	Rarely/ Never	1-3 times a month	Once a week	2-4 week	5-6 week	Once a day	2-3 day	4+ day
**Soy**								
q29p7 Soy milk	○	○	○	○	○	○	○	○
q29p8 Yogurt from soy	○	○	○	○	○	○	○	○
q29p9 Tofu	○	○	○	○	○	○	○	○

30. Cheese

q30p1 Any cheese	○	○	○	○	○	○	○	○
q30p2 Hard cheeses (e.g. Cheddar, parmesan)	○	○	○	○	○	○	○	○
q30p3 Soft cheeses (e.g. Brie, camembert, Philadelphia, tomini, boursault, brinza, chaource, [illegible]	○	○	○	○	○	○	○	○
q30p4 Semi-hard cheeses (e.g. Gouda, Emmental/Edam)	○	○	○	○	○	○	○	○
q30p5 Cottage cheese (cheese curd) (natural/with scents)	○	○	○	○	○	○	○	○
q30p6 Hard and semi-hard Greek cheeses (e.g. Kaseri, kefalotiri, Grafiera, Kefalograviera, Ladotiri)	○	○	○	○	○	○	○	○
q30p7 Fresh cheeses (e.g. Feta, mozzarella)	○	○	○	○	○	○	○	○

31. Other milk-derived products

q31p1 Ice cream	○	○	○	○	○	○	○	○
q31p2 Single cream crème	○	○	○	○	○	○	○	○
q31p3 Crème fraîche	○	○	○	○	○	○	○	○
q31p4 Sour cream	○	○	○	○	○	○	○	○
q31p5 Double or clotted cream	○	○	○	○	○	○	○	○

32. Miscellaneous food

q32p1 Dressing sauces (e.g. French, Cesar, thousand islands)	○	○	○	○	○	○	○	○
q32p2 Mayonnaise)	○	○	○	○	○	○	○	○
q32p3 White sauce	○	○	○	○	○	○	○	○
q32p4 Ketchup	○	○	○	○	○	○	○	○

	Rarely/ Never	1-3 times a month	Once a week	2-4 week	5-6 week	Once a day	2-3 day	4+ day
q32p5 Instant soup	○	○	○	○	○	○	○	○
q32p6 Pizza (any)	○	○	○	○	○	○	○	○
q32p7 Brown sauce	○	○	○	○	○	○	○	○
	○	○	○	○	○	○	○	○
	○	○	○	○	○	○	○	○
	○	○	○	○	○	○	○	○
	○	○	○	○	○	○	○	○
	○	○	○	○	○	○	○	○
	○	○	○	○	○	○	○	○
	○	○	○	○	○	○	○	○
	○	○	○	○	○	○	○	○
	○	○	○	○	○	○	○	○
	○	○	○	○	○	○	○	○
	○	○	○	○	○	○	○	○

Additional questions:

33. Products for special nutritional use

Do you REGULARLY take any nutritional supplement? e.g. vitamin C, selenium etc?

Yes ○ No ○

If you answered yes to question 33, please indicate:

Nutrient supplement (or brand name)	Dose taken	Times per week dose is taken
q33p1 __________	____	__
q33p2 __________	____	__
q33p3 __________	____	__
q33p4 __________	____	__

34. Are there any other foods you normally eat once or more a week?

Yes ○ No ○

If yes, please list below:

Food (if it is a local dish, and you know the main components or ingredients, please name them)	Usual serving size	Number of times eaten per week
q34p1 |___|___|___|___|___|___|___|___|___|___|	|___|___|___|___|___|	|___|___|
q34p2 |___|___|___|___|___|___|___|___|___|___|	|___|___|___|___|___|	|___|___|
q34p3 |___|___|___|___|___|___|___|___|___|___|	|___|___|___|___|___|	|___|___|
q34p4 |___|___|___|___|___|___|___|___|___|___|	|___|___|___|___|___|	|___|___|

35. What kind of fat did you most often use for frying, roasting, grilling, etc?

Select one only please:

Butter	◯
Lard/dripping	◯
Sunflower oil	◯
Solid vegetable fat	◯
Margarine	◯
Olive oil	◯
None	◯

36. How often do you add salt to food while cooking?

Always	◯
Sometimes	◯
Rarely	◯
Never	◯
	◯

37. In the last year, on average, how many times a week did you eat a medium serving (unit/glass or cup) of the following food groups?

Food type	Times/week
q37p1 Vegetables (excluding potatoes)	◯◯◯◯◯◯◯ 1 2 3 4 5 6 7
q37p2 Potatoes	◯◯◯◯◯◯◯ 1 2 3 4 5 6 7
q37p3 Fruits and fruit products (excluding fruit juice)	◯◯◯◯◯◯◯ 1 2 3 4 5 6 7
q37p4 Fish	◯◯◯◯◯◯◯ 1 2 3 4 5 6 7
q37p5 Fish products	◯◯◯◯◯◯◯ 1 2 3 4 5 6 7
q37p6 Meat, meat products or meat dishes (including bacon, ham and chicken)	◯◯◯◯◯◯◯ 1 2 3 4 5 6 7
q37p7 Milk (skimmed, full fat, any)	◯◯◯◯◯◯◯ 1 2 3 4 5 6 7

38. Are there any foods you do not eat because they cause you allergy or intolerance?

Yes ◯ No ◯

If yes, please name these foods below:

Food not consumed	Reason
q38p1 |___|___|___|___|___|___|___|___|___|___|	|___|___|___|___|___|
q38p2 |___|___|___|___|___|___|___|___|___|___|	|___|___|___|___|___|
q38p3 |___|___|___|___|___|___|___|___|___|___|	|___|___|___|___|___|
q38p4 |___|___|___|___|___|___|___|___|___|___|	|___|___|___|___|___|

THANK YOU FOR YOUR COOPERATION!

Appendix 2: List of list of clinicians and health care professionals at the participating centres

France: P Stroumza, C Boriceanu, M Lankester, JL Poignet, Y Saingra, M Indreies, J Santini, Mahi A, A Robert, P Bouvier, T Merzouk, F Villemain, A Pajot, F Tollis, M Brahim-Båounab, A Benmoussa, S Albitar, MC Guimont, P Ciobotaru, A Guerin, M Diaconita; Germany: M Hansis, SH Hoischen, J Saupe, I Ullmann S Grosser, J Kunow, S Grueger, D Bischoff, J Benders, P Worch, T Pfab, N Kamin, M Roesch M May; **Hungary**: M Török, K Albert, I Csaszar, E Kiss, D Kosa, A Orosz, J Redl, L Kovacs, E Varga, M Szabo, K Magyar, E Zajko, A Bereczki, J Csikos, E Kerekes, A Mike, K Steiner, E Nemeth, K Tolnai, A Toth, J Vinczene, Sz Szummer, E Tanyi, M Szilvia; **Italy**: L Gargano, AM Murgo, N Sanfilippo, N Dambrosio, C Saturno, G Matera, M Benevento, V Greco, G di Leo, S Papagni, F Alicino, A Marangelli, F Pedone, AV Cagnazzo, R Antinoro, ML Sambati, C Donatelli, F Ranieri, F Torsello, P Steri, C Riccardi, A Flammini, L Moscardelli, E Boccia, M Mantuano, R Di Toro Mammarella, M Meconizzi, R Fichera, A D'Angelo, G Latassa, A Molino, M Fici, A Lupo, G Montalto, S Messina, C Capostagno, G Randazzo, S Pagano, G Marino, D Rallo, A Maniscalco, OM Trovato, C Strano, A Failla, A Bua, S Campo, P Nasisi, A Salerno, S Laudani, F Grippaldi, D Bertino, DV Di Benedetto, A Puglisi, S Chiarenza, M Lentini Deuscit, CM Incardona, G Scuto, C Todaro, A Dino, D Novello, A Coco; **Poland**: AB Skublewsk, J Duława, E Bocheńska-Nowacka, A Jaroszyński, J Drabik, M Wypych-Birecka, D Daniewska, M Drobisz, K Doskocz, G Wyrwicz-Zielińska, A Kosicki, W Ślizień, P Rutkowski, S Arentowicz, S Dzimira, M Grabowska, J Ostrowski, A Całka, T Grzegorczyk, W Dżugan, M Mazur, M Myślicki, M Piechowska, D Kozicka; **Portugal**: V de Sá Martins, L Aguiar, AR Mira, B Velez, T Pinheiro; **Romania**: D Timofte, E Agapi, CL Ardelean, A Baidog, G Bako, M Barb, A Blaga, E Bodurian, V Bumbea, E Dragan, D Dumitrache, L Florescu, N Havasi, S Hint, R Ilies, AGM Mandita, RI Marian, SL Medrihan, L Mitea, S Mitea, R Mocanu, DC Moro, M Nitu, ML Popa, M Popa, E Railean, AR Scuturdean, K Szentendrey, CL Teodoru, A Varga; **Spain**: AG Bernat, D Del Castillo, M García, M Olaya, V Abujder, J Carreras, A López, F Ros, G Cuesta, A García, E Orero, E Ros, S Bea, JL Pizarro, S Luengo, A Romero, M Navarro, L Cermeño, A Rodriguez, D Lopez, A Barrera, F Montoya, J Tajahuerce, M Carro, MQ Cunill, S Narci, T Ballester, MJ Soler, S Traver, PP Buta, L Cucuiat, L Rosu, I Garcia, CM Gavra, R Gonzalez, S Filimon, M Peñalver, V Benages, MI Cardo, E García, P Soler, E Fernnandez, F Popescu, R Munteanu, E Tanase, F Sagau, D Prades, S Esteller, E Gonzalez, R Martinez, A Diago, J Torres, E Perez, C Garcia, I Lluch, J Forcano, M Fóns, A Rodríguez, NA Millán, J Fernández, B Ferreiro, M Otero, V Pesqueira, S Abal, R Álvarez, C Jorge, I Rico, J de Dios Ramiro, L Duzy, A Soto, JL Lopez, Y Diaz, I Herrero, M Farré, C Blasco, S Ferrás, MJ Agost, C Miracle, J

Farto; **Sweden**: E Fabricius, J Goch, KS Katzarski, A Wulcan, C Wollheim, J Hegbrant; **Turkey**: T Ecder, H Akbiber, H Arslan, L Bicen, A Buyukkiraz, R Celik, İS Dogan, S Erkalkan, A Ertas, U Hark, E Iravul, M Karakaya, K Mengu, S Ongun, Z Ozkan, A Ozlu, N Ozveren, HM Sifil, N Sonmez Turksoz, Z Yilmaz.
